# Predictive Factors for Malignancy in Atypiai of Undetermined Significance (AUS) Thyroid Nodules: A Comprehensive Retrospective Analysis

**DOI:** 10.3390/curroncol32040188

**Published:** 2025-03-24

**Authors:** Samet Şahin, Hikmet Pehlevan Özel, Yunus Nadi Yüksek

**Affiliations:** 1 School of Medicine, Muğla Sıtkı Koçman University, Muğla 48121, Türkiye; 2 Ankara Bilkent City Hospital, Ankara 06800, Türkiye; hikmet.pehlevan@gmail.com (H.P.Ö.); ynyuksek@gmail.com (Y.N.Y.)

**Keywords:** atypia of undetermined significance (AUS), thyroid nodules, malignancy prediction, calcification, papillary thyroid carcinoma (PTC), fine-needle aspiration biopsy (FNAB)

## Abstract

This retrospective study aimed to identify predictive factors for malignancy in thyroid nodules classified as atypia or follicular lesion of undetermined significance (AUS/FLUS). The analysis included 165 patients who underwent thyroid nodule surgery at Ankara Numune Training and Research Hospital. Data on demographics, surgical procedures, ultrasonographic features, and pathology results were extracted and analyzed. The cohort consisted predominantly of women (79.39%) with a mean age of 46.68 years. Surgeries performed included total thyroidectomy (88%), total thyroidectomy with central lymph node dissection (6%), and modified radical neck dissection (3%). Malignancies, largely papillary thyroid carcinoma (PTC), were identified in 81 cases. Univariate analysis revealed significant associations between malignancy and ultrasonographic features like calcification, spiculated margins, and nuclear inclusions. Multivariate analysis pinpointed calcification as the only independent risk factor. Histopathological findings indicated heterogeneity within malignancies, noting lymphovascular and capsular invasion in PTC cases. These findings emphasize calcification as a key predictor of malignancy in AUS thyroid nodules and underscore the role of surgical intervention in this challenging diagnostic category, contributing to enhanced risk stratification and clinical decision-making for managing AUS/FLUS thyroid nodules.

## 1. Introduction

Thyroid nodules are a prevalent clinical entity, often evoking concerns about malignancy. Their prevalence is substantial, with ultrasound imaging revealing their existence in 30–50% of the population [1]. Fortunately, the majority of these nodules, approximately 95%, prove to be benign [2]. Amidst this landscape, fine-needle aspiration biopsy (FNAB) emerges as a crucial diagnostic tool, offering a simple and effective means to discern between benign and malignant thyroid nodules [3]. The Bethesda system, introduced by the National Cancer Institute in 2007, provides a standardized framework for categorizing FNAB results, aiding clinicians in assessing the risk for malignancy associated with various findings [4].

A significant challenge arises with the category termed atypia or follicular lesion of undetermined significance (AUS/FLUS) within the Bethesda system. It is important to note that while the 2017 update of the Bethesda System maintains AUS and FLUS as a single diagnostic category (Category III), they represent different cytological findings that are managed similarly. Comprising 3% to 6% of all biopsies, AUS/FLUS results introduce a level of uncertainty, making it difficult to definitively classify nodules as either benign or malignant [4]. This uncertainty necessitates further investigation and clinical correlation to determine the appropriate course of action. A meta-analysis encompassing a substantial cohort of FNAB cases and subsequent surgeries reported an overall AUS/FLUS rate of 9.6%, with a 15.9% risk of malignancy [5].

The intricate nature of AUS/FLUS results prompts the need for thorough evaluation and a personalized approach to patient management. Notably, studies have reported a wide range of malignancy risks associated with this category, varying from 5% to 19% [5,6]. The challenge lies in determining the optimal strategy for patients falling under the AUS/FLUS classification. Clinical correlation, alongside repeated FNAB, is often recommended, and surgery becomes a viable option for cases presenting worrisome clinical or ultrasound findings [4,7,8,9].

A review of the literature revealed that AUS and FLUS are often discussed together, with limited studies evaluating AUS as a separate entity. In the pursuit of enhancing our understanding of AUS thyroid nodules, this study aims to evaluate the predictive value of various demographic, ultrasonographic, laboratory, and pathological characteristics. By comparing preoperative data from patients who underwent surgery for AUS thyroid nodules, we aspire to contribute valuable insights into refining risk stratification and guiding informed clinical decisions. The multifaceted nature of AUS cases necessitates a comprehensive exploration, taking into account not only the Bethesda system but also specific ultrasonographic features and other clinicopathological factors [10,11,12].

As we embark on this exploration, it is essential to recognize the existing body of literature, which has yielded diverse findings and insights into the factors influencing malignancy risk in AUS thyroid nodules. Through a comparative analysis with previous studies, we seek to contextualize our observations and contribute to the evolving understanding of this intricate clinical scenario. Moreover, this study acknowledges its limitations, including the retrospective design and single-institution focus, underscoring the need for continued research and validation of our findings in larger, prospective cohorts.

In summary, the assessment of AUS thyroid nodules demands a nuanced approach, considering the intricate interplay of various factors [4,13]. This study endeavors to shed light on the predictive value of demographic, ultrasonographic, laboratory, and pathological characteristics, aiming to enhance risk stratification and inform clinical decision-making in the dynamic landscape of thyroid nodule management.

## 2. Materials and Methods

### 2.1. Study Design

This retrospective clinical study was meticulously designed to investigate the predictive factors associated with malignancy in thyroid nodules categorized as AUS. The study encompassed a comprehensive analysis of medical records from patients who underwent thyroid nodule surgery at Ankara Numune Training and Research Hospital, Department of General Surgery, spanning the period from June 2013 to November 2016.

### 2.2. Study Population

This study included both male and female patients aged 18 years and older, specifically those whose fine-needle aspiration biopsy (FNAB) results were reported as AUS. A total of 165 patients met the inclusion criteria. Exclusion criteria involved cases where cytological findings were reported as “suspicious for malignancy”, “follicular lesion of undetermined significance”, or “malignant” upon repeated FNAB. All patients underwent thyroid function tests and ultrasonography before surgery. In accordance with the 2017 Bethesda System for Reporting Thyroid Cytopathology, we classified nodules as AUS/FLUS (Category III) based on established cytological criteria.

### 2.3. Data Collection

To ensure a comprehensive data collection process, information was extracted from various sources, including physician notes, imaging reports, pathology reports, and laboratory data integrated into the hospital automation system. Demographic data, such as gender, age, family history, and radiation history, were collected. Laboratory results, specifically thyroid-stimulating hormone (TSH) values, were recorded. Ultrasonographic findings included nodule number, nodule size, hypoechogenicity, spiculated margin, and the presence of a solid or cystic component and microcalcification. Calcifications were classified into five distinct categories based on their ultrasonographic characteristics. Microcalcifications were defined as fine stippling calcifications, often resembling psammoma bodies, while annular-like peripheral calcifications were identified as those outlining the nodule periphery in a ring-like fashion. Crescent-like peripheral calcifications were characterized by a crescent shape along a portion of the nodule’s border, and intranodular coarse calcifications were recognized as larger, irregular deposits located within the nodule. Calcified spots were defined as small, isolated calcifications that did not fit into any of the other categories. Additionally, the pathological findings of FNAB, encompassing nuclear clearing, nuclear grooving, nuclear inclusion, or irregular nuclear membrane were documented [14].

### 2.4. Surgical Procedures

Information regarding the type of surgery performed was also included in the dataset, distinguishing between total thyroidectomy (TT), total thyroidectomy with central lymph node dissection (TT + CLND), and total thyroidectomy with modified radical neck dissection (TT + MRND). The surgical procedures were categorized based on the latest pathology results, ultimately classifying patients into benign or malignant groups. The malignant group further specified the types of malignancies, including papillary thyroid carcinoma (PTC), follicular thyroid carcinoma (FTC), and tumors of uncertain malignant potential.

### 2.5. Statistical Analysis

To draw meaningful conclusions from the collected data, statistical analyses were conducted using SPSS Statistics version 18.0 (SPSS Inc., Chicago, IL, USA). Categorical variables, such as atypical descriptors, ultrasonography criteria for malignancy, and gender, were analyzed using the chi-square test. Continuous variables, including age, nodule size, and number, were subjected to *t*-tests. Multivariate logistic regression with a backward stepwise variable selection procedure was employed to identify variables associated with an increased risk of malignancy. Variables showing a significance level (*p*-value) less than 0.05 in the univariate analysis were incorporated into the multivariate model, while non-significant variables were systematically removed through the backward selection procedure.

## 3. Results

### 3.1. Demographic and Clinical Characteristics

This retrospective analysis encompassed a cohort of 165 patients with AUS thyroid nodules, of whom 131 (79.39%) were female and 34 (20.61%) were male. The mean age of the study population was 46.68 (±11.78) years. Notably, a mere four patients reported a family history of thyroid disorders, while one patient had a history of radiation exposure.

### 3.2. Surgical Procedures and Pathological Outcomes

Among the 165 patients, 148 (88%) underwent total thyroidectomy (TT), 11 (6%) underwent TT with central lymph node dissection (CLND), and 6 (3%) underwent TT with modified radical neck dissection (MRND). Following the latest pathology results, patients were categorized into benign (84 patients) and malignant (81 patients) groups. Within the malignant group, the specific malignancies were identified, with 70 cases of papillary thyroid carcinoma (PTC), 2 cases of follicular thyroid carcinoma (FTC), and 9 cases classified as tumors of uncertain malignant potential (Table 1).

### 3.3. Ultrasonographic Findings

The comparative analysis of ultrasonographic findings between benign and malignant groups revealed significant distinctions. Univariate analyses highlighted the statistical significance of calcification occurrence, spiculated margin, and nuclear inclusion in the malignant group (*p* = 0.032, *p* = 0.025, and *p* = 0.038, respectively) (Table 2). However, other ultrasonographic parameters did not exhibit statistically significant differences between the two groups.

### 3.4. Diagnostic Performance of Calcifications

This analysis revealed that the presence of any calcification in thyroid nodules had a sensitivity of 85.2%, a specificity of 48.8%, and a positive predictive value of 61.6%, with an odds ratio of 5.48 (*p* < 0.001), indicating a statistically significant association with malignancy (Table 3). In contrast, individual calcification subtypes such as annular, crescent, calcified spot, and intranodular patterns did not show significant associations (*p* > 0.05). Notably, microcalcifications demonstrated moderate sensitivity (49.4%) and specificity (73.8%) with an odds ratio of 2.75 (*p* = 0.002), supporting their role as a significant predictor of malignancy.

### 3.5. Multivariate Analysis

Subsequent to univariate analyses, multivariate logistic regression was employed to discern independent predictors of malignancy. Intriguingly, in the multivariate analysis, only the presence of calcification emerged as an independent risk factor for malignancy, unlike the broader range of significant variables identified in the univariate analyses (Table 4).

### 3.6. Histopathological Characteristics

Histopathological examination of malignant cases further delineated the nature of malignancies. Among the PTC cases, 4 exhibited lymphovascular invasion, and 21 demonstrated capsular invasion, underscoring the diverse pathological characteristics within the malignant subset.

## 4. Discussion

### 4.1. Previous Studies and Divergent Findings

Navigating the intricacies of AUS in thyroid nodules requires a comprehensive understanding of previous studies. Kuru et al.’s identification of diverse risk factors, ranging from solid structure to microcalcification, initiates our exploration into the complex landscape of AUS cases [15]. However, as we delve further, we encounter divergent findings, necessitating a nuanced examination of clinicopathological factors that contribute to the heterogeneity in conclusions [16,17]. Hong et al.’s report on spiculated margins, nuclear grooving, and irregular nuclei adds another layer to the intricate puzzle, prompting us to dissect these variations and draw comprehensive insights [18].

### 4.2. Ultrasonographic Findings

In the realm of AUS thyroid nodules, uncovering the impact of various clinicopathological factors demands a meticulous examination. As we scrutinize gender, age, and ultrasonographic findings, including nodule size, echogenicity, and calcification occurrence, a nuanced narrative unfolds. The initial exploration reveals significant differences in univariate analyses, particularly in calcification, irregular margin, and nuclear inclusion between benign and malignant groups. However, the multivariate analysis refines our understanding, highlighting the pivotal role of calcification as the solitary independent risk factor for malignancy in AUS cases.

### 4.3. Calcification

Our analysis revealed that the overall presence of calcification in thyroid nodules is a significant predictor of malignancy, as evidenced by a high sensitivity of 85.2% and an odds ratio of 5.48 (*p* < 0.001). Among the individual calcification subtypes, microcalcifications were particularly notable, demonstrating a sensitivity of 49.4%, a specificity of 73.8%, and an odds ratio of 2.75 (*p* = 0.002), thereby reinforcing their established role as a marker for malignancy. In contrast, annular, crescent, calcified spot, and intranodular calcifications did not reach statistical significance, suggesting that these patterns may have limited utility as independent predictors in our cohort. These findings underscore the importance of evaluating calcification types comprehensively, as the overall presence of calcification and specifically microcalcifications appear to carry significant diagnostic implications for risk stratification in thyroid nodule evaluation.

### 4.4. Comparison with Literature

To contextualize our findings, we draw parallels with existing literature, forming a cohesive narrative that underscores the significance of our study. Aligning with previous studies, our observations point to consistent associations between certain ultrasonographic features—calcification and spiculated margin—and malignancy risk in AUS cases [19]. This alignment not only validates our findings but also emphasizes the need for corroborative evidence in strengthening the understanding of these factors. As we navigate through this comparative analysis, the synthesis of knowledge offers a robust foundation for interpreting the clinical implications of our study.

In line with our findings, Kim et al. demonstrated that ultrasonographic microcalcification (MC) is highly associated with papillary thyroid cancer and, importantly, that US MC-type calcification is consistently related to the presence of pathologic psammoma bodies, a well-established poor prognostic factor. Their retrospective review of 1297 thyroid nodules revealed that while all nodules exhibiting US MC-type calcification contained psammoma bodies, non-MC calcifications were more often associated with ossification, with stromal calcifications distributed across both categories. These results reinforce the concept that detailed assessment of calcification patterns—specifically the identification of MC on ultrasound—can serve as a valuable prognostic indicator of aggressive disease in PTC, supporting its use in preoperative risk stratification and clinical decision-making [14].

Lee et al. reported that while microcalcifications suggest malignancy, macrocalcifications lack diagnostic reliability [20]. In their study of thyroid nodules with macrocalcification, ultrasound-guided FNA demonstrated a high diagnostic yield with 98.51% sensitivity, 90.91% specificity, and an overall accuracy of 96%, and no significant differences in malignancy risk were observed among the macrocalcification subtypes (smooth total, smooth partial, irregular, and nodular; *p*  >  0.05). Similarly, the multi-institutional study by Rossi et al. revealed that psammoma bodies, traditionally considered hallmark features of papillary thyroid carcinoma, can also be encountered in benign thyroid lesions such as thyroid follicular nodular disease, Hashimoto thyroiditis, and follicular adenomas [21]. These findings emphasize that while certain calcification patterns, notably microcalcifications, are important predictors of malignancy, the presence of macrocalcifications or psammoma bodies alone should be interpreted with caution, necessitating thorough histopathologic evaluation to exclude occult carcinoma.

Many calcification types are defined in the literature, emphasizing their diagnostic implications. Lacout et al. highlighted that thyroid calcifications, including both microcalcifications and macrocalcifications, are observed in benign and malignant thyroid conditions, with microcalcifications being particularly significant in papillary thyroid carcinoma (PTC) due to their association with psammoma bodies [22]. Specifically, microcalcifications, reflecting calcified tumor papillae, frequently correlate with malignancy and metastatic potential. Conversely, macrocalcifications, particularly those exhibiting complete “eggshell” patterns, are often associated with benign nodules unless interrupted or irregular, suggesting potential malignant infiltration. In our study, we similarly found that calcification, specifically the presence of microcalcifications, was significantly associated with malignancy in thyroid nodules classified as AUS/FLUS. Consistent with Lacout et al., our findings reinforced microcalcifications as a critical ultrasound marker for predicting malignancy, evidenced by a sensitivity of 49.4%, specificity of 73.8%, and a statistically significant odds ratio of 2.75 (*p* = 0.002). However, unlike Lacout et al., who detailed various patterns of macrocalcifications and their variable diagnostic reliability, our data did not demonstrate significant associations between malignancy and other macrocalcification subtypes such as annular, crescent-shaped, or intranodular patterns. This discrepancy underlines the importance of careful interpretation and the necessity for complementary diagnostic approaches, particularly in cases with ambiguous ultrasound features.

Differences in calcification patterns between studies may stem from underlying molecular mechanisms driving their formation. Ferreira et al. emphasized that microcalcifications, particularly psammoma bodies, are closely linked to malignancy due to their association with osteogenic signaling pathways involving molecules like osteopontin, Runx-2, and alkaline phosphatase [23]. These molecules facilitate hydroxyapatite deposition, primarily through macrophage-derived matrix vesicles, contributing to the malignant transformation of thyroid nodules. In contrast, our study, while confirming the strong predictive value of calcifications for malignancy, did not differentiate between molecular subtypes of calcifications. The variation in the predictive power of different calcification types—microcalcifications being highly associated with malignancy, whereas other forms such as coarse or rim-like calcifications having inconsistent significance—may be attributable to differences in the molecular processes governing their formation. This suggests that not all calcifications observed on ultrasonography have equal pathological relevance and highlights the potential role of molecular markers in refining thyroid cancer risk stratification.

Recent advances in deep learning (DL) have also begun to influence the diagnostic process for thyroid nodules. Chen et al. (2023) demonstrated that DL models, such as the Xception network, can outperform radiologists in distinguishing benign from malignant calcified thyroid nodules [24]. We propose that such DL methods may serve as valuable adjunctive tools, complementing traditional ultrasonographic assessments and further refining the risk stratification, especially in AUS cases where diagnostic ambiguity is high.

In the 2023 update to the Bethesda classification, the distinction between AUS (Atypia of Undetermined Significance) and FLUS (Follicular Lesion of Undetermined Significance) in Category III has been unified under a single definition [25]. However, because our data were collected between 2013 and 2016, we did not have the opportunity to apply this new nomenclature to our sample. This represents one of the limitations of our study, and future research should consider incorporating the most current Bethesda guidelines.

### 4.5. Study Limitations and Implications

Acknowledging the constraints of our study is pivotal for a comprehensive interpretation of its contributions. In the exploration of clinicopathological factors influencing malignancy risk in AUS thyroid nodules, the retrospective nature, single-institution focus, and relatively small sample size loom as limitations. As we delve into these limitations, we recognize their potential impact on the generalizability of our findings. The inclusion of solely AUS patients introduces a specific context, prompting us to reflect on the potential selection bias and emphasizing the need for broader, prospective studies. Despite these constraints, our study lays the groundwork for future research, urging a critical examination of the intricacies of AUS thyroid nodules.

We identify calcification as an independent risk factor for malignancy in AUS nodules, distinguishing it from other ultrasonographic and cytologic features that showed significant associations in univariate analysis but did not retain independent predictive value in multivariate analysis. The strong predictive role of calcifications, particularly microcalcifications, in our cohort aligns with prior literature on thyroid malignancy while highlighting its specific relevance within AUS/FLUS cases, a subset that has not been thoroughly evaluated in previous calcification studies. The observed statistical significance of calcifications suggests that their presence should carry substantial weight in clinical decision-making for AUS cases, where malignancy risk assessment remains a significant challenge.

## 5. Conclusions

In the dynamic landscape of managing AUS thyroid nodules, our study serves as a compass, offering critical insights into clinicopathological factors that warrant consideration in clinical decision-making. The identification of calcification as an independent risk factor for malignancy prompts a re-evaluation of specific ultrasonographic features. As we navigate through the concluding remarks, our nuanced perspective reinforces the importance of tailoring clinical decisions based on unique histological and ultrasonographic characteristics. This conclusion not only summarizes our findings but also underscores the ongoing necessity for research to refine guidelines in the management of AUS thyroid nodules.

## Figures and Tables

**Table 1:** Surgery types of 165 patients with AUS.

	TT	TT + CLND	TT + MRND	TOTAL
Benign	82	2	0	84 (51%)
PTC	56	8	6	70 (42.4%)
FTC	2	0	0	2 (1.2%)
Tumor of uncertain malignant potential	8	1	0	9 (5.4%)
TOTAL	148	11	6	165

PTC: papillary thyroid carcinoma; FTC: follicular thyroid carcinoma; TT: total thyroidectomy; CLND: central lymph node dissection; MRND: modified radical neck dissection.

**Table 2:** Relationship of final pathological results (benign or malignant) with clinicopathological factors.

Parameters		Benign	Malignancy	*p*-Value
Age (±SD)		47.24 ± 11.987	46.10 ± 11.60	0.536
Gender	Female	68 (80.9%)	63 (64.9%)	0.614
	Male	16 (19.1%)	34 (35.1%)	
TSH (mg/dl) (Median (IQR 25–75))		0.93 (0.36–2.77)	1.22 (0.72–1.77)	0.216
Number of nodules (US) (Median (IQR 25–75))		3 (2–6)	4 (2–6)	0.879
Size of nodule mm (US) (Median (IQR 25–75))		15 (11–28)	15 (10–24)	0.770
Hypoechogenecity (US)	(+)	21 (25%)	26 (32.1%)	0.531
	(−)	63 (75%)	55 (67.9%)	
Cystic/solid state (US)	Cystic	18 (21.5%)	15 (18.5%)	0.474
	Solid	58 (69%)	56 (69.2%)	
	Mixt	8 (9.5%)	10 (12.3%)	
Calcification (US)	(+)	22 (26.2%)	39 (48.1%)	0.032 *
	(−)	62 (73.8%)	42 (51.9%)	
Spiculated Margin (US)	Regular	70 (84.3%)	50 (60.9%)	0.025 *
	Irregular	13 (15.7%)	32 (39.1%)	
Nuclear Clearing (FNAB)	(+)	11 (13.1%)	13 (16.1%)	0.591
	(−)	73 (86.9%)	68 (83.9%)	
Nuclear Grooving (FNAB)	(+)	26 (30.9%)	34 (41.9%)	0.141
	(−)	58 (69.1%)	47 (58.1%)	
Nuclear Inclusion (FNAB)	(+)	5 (27.8%)	79 (53.7%)	0.038 *
	(−)	13 (72.2%)	68 (46.3%)	
Nuclear Membrane (FNAB)	Regular	46 (54.8%)	40 (49.4%)	0.489
	Irregular	38 (45.2%)	41 (50.6%)	

* *p* < 0.05 is statistically significant (US: ultrasonography findings; FNAB: cytological findings with fine needle aspiration biopsy).

**Table 3:** Diagnostic performance metrics of thyroid nodule calcification patterns.

Calcification Type	Sensitivity (%)	Specificity (%)	PPV (%)	Odds Ratio	*p* Value
Any Calcification	85.2	48.8	61.6	5.48	<0.001 *
Annular	3.7	97.6	60.0	1.58	0.483
Crescent	7.4	95.2	60.0	1.60	0.530
Calcified Spot	8.6	94.0	58.3	1.49	0.506
Intranodular	14.8	88.1	54.5	1.29	0.583
Microcalcification	49.4	73.8	64.5	2.75	0.002 *

* *p* < 0.05 is statistically significant.

**Table 4:** Multivariate analysis of factors associated with malignancy.

	95% CI	*p*-Value
Calcification (US)	2.033 (1.072–3.857)	0.030 *
Spiculated Margin (US)	1.82 (0.918–3.608)	0.086
Nuclear Inclusion (FNAB)	2.86 (0.941–8.692)	0.064

* *p* < 0.05 is statistically significant (CI: confidence interval; US: ultrasonography findings; FNAB: cytological findings with fine needle aspiration biopsy).

## Data Availability

No data are available due to privacy or ethical restrictions.
